# Everybody's Page

**Published:** 1890-08-30

**Authors:** 


### Everybody's Page.

The death-rate of the sailors in the mercantile marine is 12 per thousand—lower than on land.

"Medical science has made such progress," said the doctor when speaking of his profession, "that it is almost impossible for anybody to be buried alive now." Then he wondered why everybody laughed.

A new and popular development of electrical science is the electrical hair curler. It is said to be equal to the most exacting demands of the feminine coiffure, and the beard or moustache can be curled in any style in two minutes. There is some good in electricity after all!

Here is an American conversation: *Health Officer*: "Have you ever been inoculated?" *Sam* (rather apologetically): "Free times, boss; da's all." *Health Officer*: "What for?" *Sam*: "Wonst for stealin' chick'ns, wonst for 'sault 'n batt'ry, an' when I jined de Masons."

Giants are usually deficient in brain-power, the sole exception known being the Irish giant, whose skeleton is preserved in Dublin, and whose skull is in proportion to the rest of his frame; dwarfs, on the other side, have often been famed for their extraordinary acuteness. There is, then, some foundation in science for the old legends in which the superior intelligence of the dwarf overcomes the strength of the giant; Jack-the-giant-killer is not so unnatural a phenomenon as some old wiseacres have thought. In technical language, a human being more than 7 ft. high is a giant, or one shorter than 4 ft. is a dwarf.

We are coming to a nice era in the care of children, and some of us can devoutly thank Providence that, in our young days, a child was subject of parental and not Government control. A little girl, aged 10, rides away from Peterborough on a bare-backed horse which she has taken from a field. The plucky youngster was out all night, sleeping under a hedge, and off next day again in search of London and its streets paved with gold. The magistrates affirmed that the child stole the horse, and they sentenced her to three weeks' imprisonment and five years in a reformatory. The father acquiesced in this terrible punishment. A small boy, aged 4½, named Noakes, pushed another boy into the water last week, and the child was drowned. The coroner has written to the Home Secretary for special powers to control Noakes, aged 4½. Now turn to the other side of the question; a labourer at Manchester thrashes his little sister with a strap for neglecting to wake him at a proper hour; the man is had up by the Society for the Prevention of Cruelty to Children, and is sentenced to a months' hard labour. Doubtless the punishment was brutal for a girl, but if sharp it was short. Surely those five years of reformatory for a spirited, active child, is far greater cruelty? No Scotch child ever attained to years of discretion without several severe "skelpings" with a strap, which is after all a safe enough instrument of punishment. In after years a joke is made between father and son about these same thrashings. But can five years' confinement—five years of beating the restless child-soul against stringent rules—five years of monotony which crushes out all individuality—can that ever be regarded as a joke? The Society for the Prevention of Cruelty to Children does well to investigate baby-farms, child insurance, and systematic ill-treatment, but it would be wise to occasionally close its eyes when a wrathful parent is cruelly severe if a few bruises are the only result; the rage of a father may be kindness compared to the calculating discipline of the Government.

The recent census enumerators have depopulated the American cities more rapidly than any epidemic.

Mothers may be glad to know that if they add alum to the blue water in which their children's clothes are rinsed after washing, it will greatly prevent these clothes catching fire should the children put themselves in danger of such an accident.

The man missionary is accused of having killed his converts by introducing them to rum; the lady missionary is now accused of causing the death of the Maori women of New Zealand, who are trying to wear corsets in imitation of their teachers. Oh the barbarities of civilization!

Cleanliness might be supposed to aid in prolonging life, yet a Mrs. Lawson, who died in the early part of this century, age 106, must have been a singularly dirty woman. We are told that instead of washing she smeared her face with lard, and asserted that "people who washed always caught cold."

The Greek boy was properly trained. Gymnastics was a no less important part of his education than grammar or music. His pedagogue marched him off to the palaestra as early as to school, and with wrestling, running, and jumping his physical discipline began. The course of instruction followed in the palaestra was consciously designed to produce beauty of form and limb. He was not allowed to develop one part of his body at the expense of the rest; he was not allowed only to ride, or only to run, or only to box; nor was he allowed to develop his body at the expense of his brain; his education was carefully calculated to make the most of all the gifts nature had bestowed on him.

About the year 1765 Madame Necker came to Paris. She was a very charitable and humane person, and set herself, among other things, to redress the evils going on in the hospitals and other social institutions of Paris. She was the first to enlist the assistance of sisterhoods in the charitable works she carried on, and this was the statement of what she saw: "At the period when she first came to the capital priding itself on every refinement of luxury, sufferers in hospitals were not even given separate beds, but as they arrived . . . indifferently shared a bed with those on the road to recovery and those who were dangerously ill, the sick or the dying, a patient with an infectious disease, or another merely temporarily indisposed. The most frightful scenes and fatal results were the daily consequence of this state of things in the dreaded refuges provided for earthly sufferings."

One of the most pathetic essays ever written by a child was that of Harry Sharman on "The Doctor." The poor little author died two weeks later from brain fever, and certainly he was the victim of over-pressure. Here are a few of his remarks:—"Being a doctor is a very good trade. Doctors have most always nice black wiskers at the side, and are tall men. They are also very fierce looking, but they are very useful. Doctors are men who never walk, except from a carriage to a house door. Doctors are skinny men, with black eyes and coats. Doctors bring babies to good little boys' houses. I was very good, and he brought my mother ours. It is a little girl, and it is called Agnes. When I'm in bed I can't sometimes go to sleep. I can say my money tables best in bed. I dreamed one night that the doctor came upstairs all in the dark, and took me out of bed, and gave me to a perlice to bury. But I woke up just afore he buried me, and my mother was akissin me and cryin. Mother says doctors can cure nearly all things, and that they are kind men. Headaches is not dangerous."

